# Diversity-Stability Dynamics of the Amphibian Skin Microbiome and Susceptibility to a Lethal Viral Pathogen

**DOI:** 10.3389/fmicb.2019.02883

**Published:** 2019-12-20

**Authors:** Xavier A. Harrison, Stephen J. Price, Kevin Hopkins, William T. M. Leung, Chris Sergeant, Trenton W. J. Garner

**Affiliations:** ^1^Institute of Zoology, Zoological Society of London, London, United Kingdom; ^2^Centre for Ecology and Conservation, University of Exeter, Exeter, United Kingdom; ^3^UCL Genetics Institute, University College London, London, United Kingdom

**Keywords:** amphibian conservation, microbiome stability, host-microbe interactions, amphibian disease, ranavirus, FV3-like ranavirus

## Abstract

Variation among animals in their host-associated microbial communities is increasingly recognized as a key determinant of important life history traits including growth, metabolism, and resistance to disease. Quantitative estimates of the factors shaping the stability of host microbiomes over time at the individual level in non-model organisms are scarce. Addressing this gap in our knowledge is important, as variation among individuals in microbiome stability may represent temporal gain or loss of key microbial species and functions linked to host health and/or fitness. Here we use controlled experiments to investigate how both heterogeneity in microbial species richness of the environment and exposure to the emerging pathogen *Ranavirus* influence the structure and temporal dynamics of the skin microbiome in a vertebrate host, the European common frog (*Rana temporaria*). Our evidence suggests that altering the bacterial species richness of the environment drives divergent temporal microbiome dynamics of the amphibian skin. Exposure to ranavirus effects changes in skin microbiome structure irrespective of total microbial diversity, but individuals with higher pre-exposure skin microbiome diversity appeared to exhibit higher survival. Higher diversity skin microbiomes also appear less stable over time compared to lower diversity microbiomes, but stability of the 100 most abundant (“core”) community members was similar irrespective of microbiome richness. Our study highlights the importance of extrinsic factors in determining the stability of host microbiomes over time, which may in turn have important consequences for the stability of host-microbe interactions and microbiome-fitness correlations.

## Introduction

Animals are host to diverse communities of microbes, collectively referred to as the microbiome. Variation among individuals in their microbiomes has been linked to variation in host resistance to pathogens (Ford and King, 2016; King et al., 2016; Villarino et al., 2016; Antwis and Harrison, 2018; Warne et al., 2019), and disruption of the microbiome by external stressors (e.g., antibiotics) can have long term negative effects on host health (Theriot et al., 2014; Knutie et al., 2017; Warne et al., 2019). Though there is growing evidence that perturbation of the microbiome can have deleterious effects on host physiology, an understanding of the drivers of individual microbiome dynamics over time, and resistance to perturbation, remain relatively scarce in non-model organisms (Loudon et al., 2014b, 2016; Videvall et al., 2019). Addressing this shortfall in our knowledge is of fundamental importance to understanding the adaptive value of microbiomes for host health and fitness, as microbiome-health correlations may not be stable over time if microbiome flux represents loss of key microbial species and/or genes critical for optimal host physiology. Variation among individuals in their resistance to microbiome perturbation, and resilience following perturbation, could be a critical determinant of the distribution and stability of traits such as resistance to pathogens in natural populations.

The amphibian skin microbiome is rapidly becoming established as a model system for understanding the tripartite relationships between host, microbiome, and pathogens (e.g., Harris et al., 2009; Longo et al., 2015; Kueneman et al., 2016; Bates et al., 2018; Campbell et al., 2018a, b, 2019; Ross et al., 2019). Production of metabolites by skin-associated bacteria is a crucial component of immune defense against lethal fungal pathogens such as *Batrachochytrium dendrobatidis* (*Bd*) (e.g., Brucker et al., 2008; Becker et al., 2009) and *Batrachochytrium salimandrivorans* (Muletz-Wolz et al., 2017). Anti-fungal metabolite production by bacteria increases dramatically when the bacteria are co-cultured (Loudon et al., 2014a), suggesting that microbiome-mediated host protection is likely a function of synergistic interactions among community members. Greater microbiome diversity may therefore offer increased protection from pathogens (e.g., Piovia-Scott et al., 2017; Antwis and Harrison, 2018; Greenspan et al., 2019; but see Becker et al., 2019), but the ecological processes structuring and maintaining microbial diversity on amphibian skin remain relatively understudied, especially at the level of the individual (Loudon et al., 2014b, 2016; Longo and Zamudio, 2017; Hughey et al., 2019). For example, the diversity-stability hypothesis predicts that more diverse communities should be more resistant to disturbance, and several empirical studies support this hypothesis in plant community assemblages (McCann, 2000; Costello et al., 2012), but it is unclear whether this theory is also relevant at the scale of host-associated microbial communities (Costello et al., 2012, but see Koskella et al., 2017). Though several studies have sought to measure the influence of pathogenic infection on host microbiome structure (Jani and Briggs, 2014; Longo et al., 2015; Longo and Zamudio, 2017), investigations of whether the magnitude of microbiome disruption for infected hosts is modulated by initial microbiome state remains relatively scarce (see Jani et al., 2017; Jani and Briggs, 2018).

Here, we use experiments to examine how both the diversity of the environmental microbial reservoir and exposure to the lethal pathogen ranavirus influence skin microbial community dynamics in a native United Kingdom amphibian species, the European Common frog (*Rana temporaria*). The emerging infectious disease (EID) ranavirosis represent a significant threat to ectothermic vertebrate health, and infection with ranaviruses is associated with mass mortality, population extirpations and declines in biodiversity at a global scale (Jancovich et al., 2005; Fox et al., 2006; Bigarre et al., 2008; Ariel et al., 2009; Une et al., 2009; Whittington et al., 2010; Jensen et al., 2011; Allender et al., 2013; Earl and Gray, 2014; Price et al., 2014, 2019; Stark et al., 2014; Brunner et al., 2015; George et al., 2015; Miaud et al., 2016; Rijks et al., 2016; Rosa et al., 2017). Ranavirus was responsible for multi-species amphibian declines in continental Europe (Price et al., 2014), and of the common frog in the United Kingdom (Teacher et al., 2010), but also alters the age structure of remnant United Kingdom common frog populations (Campbell et al., 2018a). The frequency and severity of disease outbreaks are predicted to worsen alongside human-mediated range expansion of ranaviruses (Jancovich et al., 2005; Schloegel et al., 2009; Price et al., 2016, 2019). To manipulate environmental microbiome diversity, we assembled experimental units that either contained a complex natural bacterial reservoir (*complex* habitats, containing a soil substrate and leaf litter) or simplified ones (*simple* habitats, containing stony terrestrial substrates and no leaf litter). We performed two sequential experiments. In the first experiment, we group-housed 96 *R. temporaria* metamorphs in blocks of six individuals (*n* = 48 individuals per habitat treatment). For the second experiment, we individually housed 48 individuals in habitat treatments (*n* = 24 per habitat). Detailed experimental protocols are listed in the section “Materials and Methods” below. Both experiments allowed us to measure the influence of environmental microbiome on host microbiome structure and disruption of the host microbiome by pathogen exposure. Experiment 1 was designed to allow us to measure habitat-dependent mortality following exposure to ranavirus. Conversely, individual housing of frogs in Experiment 2 allowed us to track individual habitat- and pathogen-dependent microbiome trajectories over time, as well as within-individual changes in microbiome stability. Specifically, we sought to test whether (i) more diverse environmental bacterial reservoirs elicited more diverse frog skin microbiomes, (ii) more diverse skin microbiomes were more stable over time; and (iii) whether microbiome diversity predicted differences in resistance to ranavirus, manifesting as lower infection burdens and/or higher survival following exposure.

## Materials and Methods

### Ethics Statement

All experimental procedures and husbandry methods were approved by the ZSL Ethics Committee before any work was undertaken and was done under licensing by the United Kingdom Home Office (PPL 70/7830, P8897246A). Animal health and welfare was monitored daily during both the rearing and experimental periods and all animals were fed *ad libitum* (Tetra Tabimin for tadpoles, small crickets dusted with calcium and the Vetark Nutrobal vitamin supplement for metamorphosed frogs) throughout.

### Experimental Protocols

#### Animal Rearing

*Rana temporaria* metamorphs were reared from tadpoles hatched from clutches sourced from United Kingdom garden ponds where ranavirosis had not been reported to the Garden Wildlife Health project^1^. Animals that completed metamorphosis were cohoused in large groups (no more than 30 per enclosure) in 460 × 300 × 170 mm Exo Terra Faunaria containing cleaned pea gravel, a large, cork bark cover object and sloped to accommodate a small aquatic area. Experimental animals were haphazardly selected from four group enclosures.

#### Preparation of Habitat Treatment Enclosures

The general layout of both habitat types was shared in that they both contained a filled, plastic PCR tip box (terrestrial platform) with a cover object, elevated above an aquatic area filled with aged tapwater and autoclaved pea gravel formed into a slope leading from the aquatic area to the platform. The two key differences were that; (i) the terrestrial platforms in complex habitats contained garden compost as a substrate, whilst the terrestrial platforms in simple habitats contained standard and autoclaved pea shingle and; (ii) leaf litter collected from Regents Park, London, was added to the aquatic area in the complex habitats. Complex habitat enclosures were left uncovered and outdoors for 2 weeks prior to the start of experiments, while simple habitat enclosures were prepared the day before frogs were transferred into replicates. During the experiment, uneaten cricket corpses were removed from simple habitat enclosures, but left in complex habitat enclosures. Experiment 1 comprised 16 replicate blocks, each housing six recently metamorphosed frogs (8 blocks/48 frogs per habitat treatment). Experiment 2 comprised 48 smaller units each housing an individual frog (24 frogs per habitat treatment). Following rearing in an outdoor facility, animals were moved to a procedure room and housed individually for 7 days in Perspex boxes with a cover object and damp paper towel as substrate to acclimatize to experimental conditions prior to any manipulations. Individuals were randomly assigned to experimental replicates and treatments (complex or simple habitats) using a script written in R (R Core Team, 2019). We note that our habitat manipulation altered both the bacterial richness in the environment and the structural composition of habitats (e.g., pea shingle vs. soil as a terrestrial substrate). Though this could have influenced the results, for example by changing the dynamics of host contact with the environment, these differences may be more representative of natural variation in microbiome-habitat relationships, where we would expect habitat heterogeneity to covary with microbiome structure.

#### Swabbing Protocols

For both experiments, we rinsed individuals in sterilized aged tap water to remove transient environmental microbes, and then swabbed the skin of the body and limbs of frogs with MW100 DrySwabs (Medical Wire Equipment, United Kingdom). In experiment 1, all animals were swabbed on Day 1 immediately preceding transfer to experimental units, then again on day 14, the latter referred to as the “pre-exposure” swab. Following the day 14 swab, we exposed individuals to either ranavirus or the control (see protocol below), and then swabbed all individuals again on Day 17 to measure the effect of ranavirus exposure (“post-exposure swab”). We swabbed all animals alive at the end of the experiment on Day 30, but do not include these data here as sample size per habitat-treatment group combination was low and unbalanced. For experiment 2, individuals were swabbed more frequently at Day 1, Day 7, Day 14 (pre-exposure), and Day 16 (post-exposure). For experiment 1 we present the pre- and post-exposure swabs as a 2-level time variable, whereas for experiment 2 we present all four time points as a time series.

Environmental swab samples (two per experimental unit, one terrestrial and one aquatic) were also collected on day 14 preceding pathogen exposure procedures. Terrestrial swabs were taken by running the swab over the terrestrial substrate and inside the cover objects twice. Aquatic swabs were taken by submerging the swab in the aquatic portion of the tank. These swabs allow us to assess how environmental microbiome diversity influences host skin microbiome diversity.

#### *Ranavirus* Exposure

Experimental units were randomly assigned to pathogen treatment group (ranavirus or sham) for both experiments using a script written in R. Prior to this, *Ranavirus* (FV3-like isolate RUK13, Cunningham et al., 2007) was cultured in EPC cells at 27°C, harvested after the cell layer had completely cleared, subjected to three rounds of freeze-thaw and then cleared of cells and cellular debris by centrifugation at 800*g* for 10 min and discarding the cell pellet. Virus titer was estimated using a 50% Tissue culture Infective Dose assay (TCID_50_) and calculated following the method of Reed and Muench (1938). Sham exposure media was produced by harvesting the supernatant of a pure culture of EPCs after the same 800*g*, 10 min spin. For exposures, animals were transferred either as co-housed groups (Experiment 1) or individually (Experiment 2) to 90 mm petri dishes containing 19 mL of aged tap water. Depending on treatment, either 1 mL of stock virus culture at 2 × 10^6^ TCID_50_/mL (giving a final exposure concentration of 1 × 10^5^ TCID_50_/mL) or 1 mL of sham media was added to the petri dish. Animals were exposed in petri dishes for 6 h before being returned to their habitat treatment enclosures. We used daily health and welfare checks throughout the experiment to monitor survival rates. We also used daily checks to monitor for signs of disease commonly associated with ranavirosis (see below: Price et al., 2016). We ended Experiment 1 on day 30 when all surviving frogs appeared physically healthy and when mortality had subsided, and Experiment 2 on day 16 following the post-exposure swab.

### 16S Sequencing and Bioinformatics

16S metagenetic library preparation was carried out using a modified version of the protocol detailed in Kozich et al. (2013) that amplifies the v4 section of the 16S rRNA gene. Sequencing was performed using 250 bp paired-end reads on an Illumina Miseq using a v2 chemistry 500 cycle cartridge (detailed information in Supplementary File “Detailed Amplicon Sequencing Methods”). Experiment 1 and 2 were processed on separate MiSeq runs, but all comparisons and statistical tests are made among samples *within* runs, so negating batch effects and inter-run variability. We processed raw 16S reads in the DADA2 pipeline (Callahan et al., 2016), using standard parameters as per the online tutorial. We used *phyloseq* (McMurdie and Holmes, 2013) for downstream sequence processing. In both experiments we removed amplicon sequence variants (ASVs) present in the no-template controls (Experiment 1: 54 ASVs of 11,640; Expt. 2: 466 of 14,963). To focus on differences in high abundance ASVs and to remove any potential bias introduced by small differences in low-abundance reads, we removed all ASVs from the dataset with fewer than 100 reads (e.g., Longo and Zamudio, 2017), leaving 5,796,063 reads of 1446 ASVs for Experiment 1, and 7,068,790 reads of 1969 ASVs for Experiment 2 used in downstream analysis. Reads per sample ranged from 6237–66993 (Experiment 1) to 16406–59607 (Experiment 2). We rarefied data to the minimum per-experiment sequencing depth prior to analysis.

### Viral Load Quantification

Liver samples were extracted with DNeasy Blood & Tissue kits (Qiagen) following the manufacturer’s protocol. We quantified viral loads in all individuals using the qPCR method of Leung et al. (2017), which normalizes viral DNA quantities relative to host DNA in the sample.

### Statistical Analysis

We conducted all statistical analyses in R. Due to differences in experimental design, the sets of analyses employed vary by experiment. For example, we did not track individual ID in group-housing in Experiment 1 and so do not examine drivers of within-individual changes in microbiome stability, but do so in Experiment 2. Likewise we did not assay survival in Experiment 2, but do present survival analyses for Experiment 1.

We fitted mixed effects models in the R package *lme4* (Bates et al., 2015) and ranked competing models by AICc using the R package *MuMIn* (Bartoñ, 2019). We considered all models within six AICc units of the best supported AICc model to have relatively equal support in the data. To remove overly complex models from consideration we also applied the nesting rule (see Richards, 2008; Harrison et al., 2018) to remove models that were more complex versions of models with better AIC support. Where we refer to the “top model set,” we refer to the delta-6-AICc model set after the nesting rule has been applied. Where appropriate, we refitted models in a Bayesian framework using the *Stan* computational framework^2^ accessed with the *brms* package (Bürkner, 2017, 2018). The advantage of the Bayesian framework is that it allows quantification of uncertainty in parameters such as slopes and *r*^2^ values. Where appropriate, we specified mildly informative priors for parameters such as the correlation between random effects and slopes to speed up sampling and optimize convergence. We assessed convergence of chains using the Gelman-Rubin statistic, and inspected plots of posterior draws to verify adequate mixing of chains and sampling. Detailed descriptions of all statistical analyses and code are provided as an R Markdown document.

#### Diversity Indices

We calculated two metrics of alpha diversity: (i) richness as the exponent of the Shannon diversity index, also referred to as the effective number of species; and (ii) evenness, measured as the Shannon index divided by the log of the number of observed sequences in a sample. To derive measures of beta diversity, we performed Non-Metric Multidimensional Scaling (NMDS) ordinations on Bray–Curtis distance among bacterial community ASV abundances distances using the R package *vegan* (Oksanen et al., 2017). We also extracted NMDS1 values from these ordinations for analysis in statistical models (see below).

#### Experiment 1

We fitted a model containing the three-way interaction among time (pre- vs. post-exposure), habitat (Complex vs. Simple) and exposure (ranavirus vs. control) as predictors of alpha diversity, with separate models for richness and evenness. All models included a random intercept term for block ID (experimental tank) and used a Negative Binomial error structure. We performed PERMANOVA analysis in the R package *vegan* to test for differences among samples in beta diversity, also containing the time:habitat:pathogen three way interaction, and marginalizing the effect of block ID.

#### Experiment 2

We fitted a model containing day, day^2^ (to permit non-linear effects of time), habitat (Complex vs. Simple) and exposure (ranavirus vs. control) as well as an interaction between day^2^ and habitat as predictors of alpha diversity. All models included a random intercept for individual, and a random slope for day given individual. More complex models could not be fitted given the data available and produced convergence warnings. We used a Negative Binomial error structure for alpha diversity models to control for overdispersion (see Harrison, 2014) and a Gaussian error structure for beta diversity models. We performed PERMANOVA on Bray-Curtis distances among samples using the R package *vegan* to test for differences in beta diversity. We fitted a model containing a 3-way interaction between habitat, day and ranavirus exposure, permutated 999 times to derive *p* values for effects. We also fitted a linear mixed effects model to examine factors predicting NMDS1 variation among individuals, and included habitat, day, day^2^ and pathogen exposure as main effects, as well as habitat:pathogen exposure, habitat:day and habitat:day^2^ as predictors. All models included a random intercept for individuals. We could not include a random slope for day given individual as this produced convergence warnings.

#### Survival Analysis

We used the R package *coxme* (Therneau, 2015) to examine differences in survival dependent on habitat and pathogen exposure whilst controlling for block ID in Experiment 1. Sample size for this analysis was 85 individuals (42 in Simple Habitats and 43 in Complex habitats) across eight habitat blocks per habitat type. We censored eight individuals because they died prior to exposure. We ranked survival models by AICc to derive a top model set.

#### Predicted Functional Analysis

We used the ASV abundance matrices from Day 7 to predict functional profiles of microbial communities using PIPHILLIN (Iwai et al., 2016) and tested for differences in functional profiles dependent on habitat using Constrained Correspondence Analysis (CCA) in the R package *vegan*. We used the May 2017 release of the KEGG database and 97% identity cutoff. We visualized differences in predicted functional repertoire by plotting the axes of a CCA model fitted in *vegan* where we specified the two-level habitat predictor as the constrained variable. We performed predicted functional analysis only on Experiment 2 data as controlling for block effects in DESeq2 (Love et al., 2014) is difficult, and individual hosing of Experiment 2 obviates the need for this and so should more tightly control the false positive rate.

#### Stability Over Time

We calculated microbiome stability as the correlation between ASV abundances across two time points, following Lahti et al. (2014). That is, microbiome stability over time is estimated as the correlation between the two vectors of microbial community abundances from an individual for two time points, where stronger correlations indicate greater stability. We used Day 7 and 13 in Experiment 2 to quantify baseline stability, and tested variation in stability dependent on habitat using a *t*-test. We also calculated stability following exposure to a pathogen using the Day 13 and Day 16 ASV abundances and tested for a correlation between pre- and post-infection stability using Spearman’s correlation tests. We also calculated change in stability across the two time points by subtracting pre-infection stability from post infection stability.

We used ANOVA to test whether change in stability was explained by habitat or pathogen treatment. We repeated the above analyses restricting the dataset to the top 100 most abundant ASVs in each habitat to represent the “core” microbiome.

## Results

### Environment and Pathogen Exposure Modify Skin Microbiome Structure (Experiment 1)

#### Alpha Diversity

**FIGURE 1:**
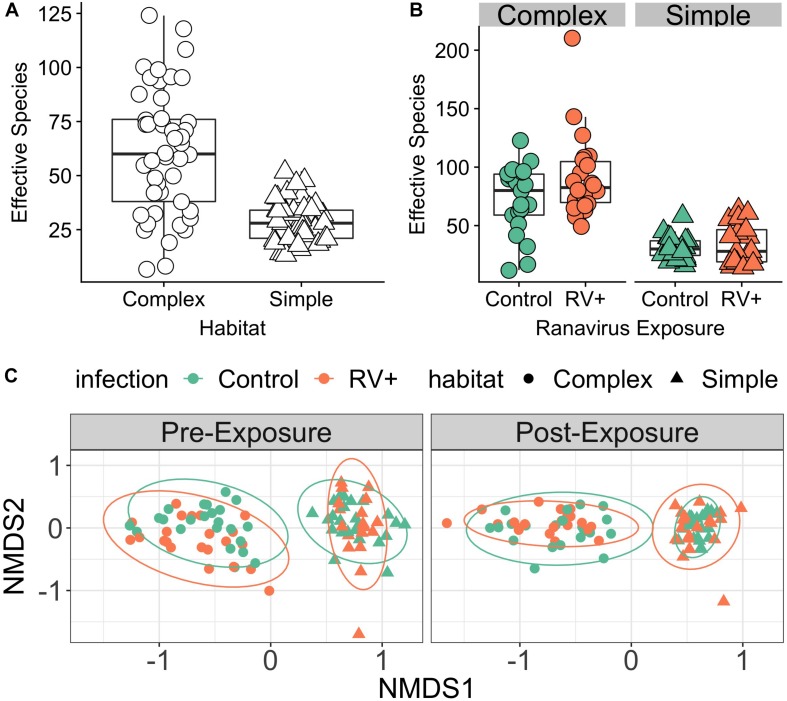
Environment modifies host skin bacterial community structure. **(A)** Effect of environmental bacterial richness on host skin microbiome diversity (Experiment 1, Time Point 2 [pre-exposure]). Individuals in habitats with more species rich environmental bacterial reservoirs also had higher skin bacterial richness (Complex) compared to individuals in habitats with lower diversity bacterial reservoirs (Simple). **(B)** There was no effect of exposure to ranavirus on mean levels of bacterial richness in either habitat type (Experiment 1, Time Point 3 [post-exposure]). **(C)** Skin bacterial community structure (beta diversity) differed significantly based on habitat and time (pre- vs. post-exposure). We detected a significant three-way interaction between time, habitat and pathogen exposure, suggesting that ranavirus exposure causes shifts in community structure dependent on habitat complexity. Note that no individuals were exposed to ranavirus in the *Pre-*Exposure panel, but individual points are colored by pathogen treatment (ranavirus vs. control) in both panels to allow comparison of groups across time.

Bacterial richness and evenness of common frog skin was directly influenced by the complexity of the bacterial species reservoir in the environment. Individuals in habitats with higher environmental bacterial species richness possessed greater mean skin bacterial diversity (*r* = 0.82, *p* = 0.001; Figure 1A and Supplementary Figure S1). There was some evidence that overall effective number of species increased over time, but only weak evidence that this effect was dependent on habitat treatment (Figure 1B and Supplementary Table S1). There was no evidence of an effect of the interaction between time, habitat and pathogen exposure, or a main effect of ranavirus exposure on overall species richness of the microbiome (Supplementary Table S1). The top model investigating drivers of differences in community evenness contained interactions between ranavirus exposure and time, as well as habitat and time (Supplementary Table S2a). As for species richness, these results indicated that community evenness was lower in Simple Habitats prior to ranavirus exposure (Supplementary Table S2b). There was also some evidence that evenness increased over time for complex habitats, but differences due to ranavirus exposure were not clear (Supplementary Figure S2 and Supplementary Table S2b).

#### Beta Diversity

PERMANOVA analysis controlling for block identified the dominant source of variation in microbial communities to be habitat (simple vs. complex, *r*^2^ = 15.9%, *p* = 0.001; Figure 1C). Exposure to ranavirus also influenced microbial community structure (infection main effect, *r*^2^ = 2.8%, *p* = 0.001), but critically operated via habitat:infection and infection:time interactions. The habitat:infection:time interaction was not significant (*p* = 0.08; Supplementary Table S3).

### Survival Following Exposure to Ranavirus (Experiment 1)

**FIGURE 2:**
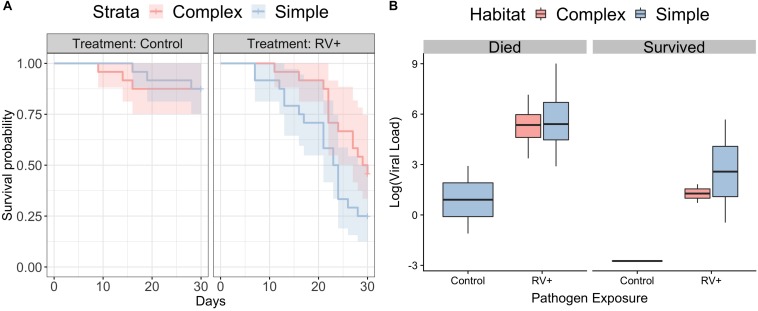
Survival following exposure to ranavirus. **(A)** Survival data for frogs exposed to Ranavirus or Control, in both Complex (red shading) and Simple (blue shading) habitats. The top model explaining variation in survival contained effects of both habitat and pathogen exposure. The model containing the habitat:pathogen interaction was not retained under the nesting rule. **(B)** Ranaviral infection loads following exposure to ranavirus, split by whether individuals died following exposure or were still alive on Day 30 at the close of the experiment. There was no difference in ranaviral infection burdens based on habitat treatment, but individuals that died had significantly higher infection loads. Three individuals in the control group exhibited weak infection.

Individuals in simple habitats exposed to ranavirus exhibited higher rates of mortality (68.4%) than individuals in complex habitats exposed to ranavirus (52.2%). The best-supported model contained effects of both habitat complexity and disease treatment on survival (Figure 2A and Supplementary Table S4). A model containing only disease treatment received marginally less support (ΔAICc = 0.22). Though the model containing the interaction between habitat and treatment was in the Δ6 AIC model set, it was a more complex version of a simpler model with better AIC support and so was removed under the nesting rule (Richards, 2008). Model averaged coefficients [and 95% confidence intervals] from the survival model were: Habitat 0.57 [−0.167,1.3] and ranavirus exposure 2.26 [1.2,3.33].

There was no difference between habitats in likelihood of exhibiting gross signs of disease (Binomial GLMM, mean probability of exhibiting signs of disease [95% credible intervals]: complex 0.48 [0.11,0.82]; simple 0.5 [0.1,0.85]; *p*_MCMC_ = 0.92) or in severity of visible signs of disease (Ordinal GLMM, mean probability of being scored category 0 [95% credible intervals]; complex 0.51 [0.12,0.9]; simple 0.46 [0.06,0.93]; *p*_MCMC_ = 0.88). Individuals that died following exposure to ranavirus had higher viral loads than those that were still alive at the end of the experiment (Figure 2B). The best supported model examining variation in viral loads contained only the main effect of mortality (*r*^2^ 38.7% [95% CI 15.1–56.1%]), as all other models with weaker support were removed under the nesting rule (Supplementary Table S5). Three individuals in the Control treatments died following exposure and exhibited weak ranavirus infections; these were inconsistent with the higher infection loads observed in other individuals that died after exposure (Figure 2B).

### Environment Alters Host Microbiome Dynamics Over Time (Experiment 2)

#### Alpha Diversity

**FIGURE 3:**
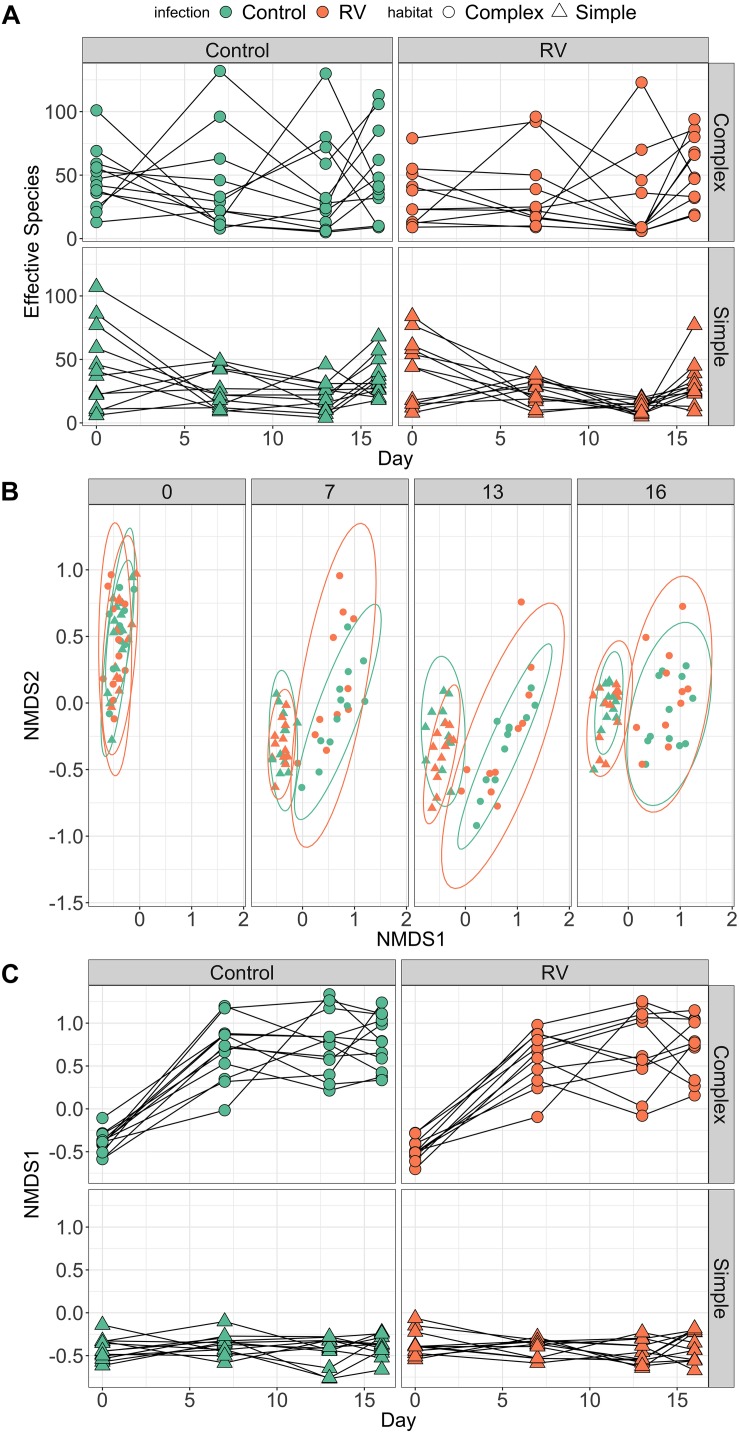
Dynamics of frog skin bacterial communities over time. **(A)** Trends over time in bacterial alpha diversity (effective number of species) depending on Habitat treatment (rows) and pathogen exposure (columns). Dynamics of alpha diversity over time were significantly different in Complex habitats. When marginalizing the effects of time (day), individuals in Complex habitats possessed higher bacterial species richness compared to individuals in Simple habitats (see Supplementary Figure S2). **(B)** Trends over time in bacterial community structure (beta diversity). Individuals were exposed to ranavirus or control between days 13 and 16, but points are colored by disease treatment at all time points to allow tracking of beta diversity over time for different groups. **(C)** Temporal trends in primary axis of NMDS ordination (beta diversity) depending on Habitat treatment (rows) and pathogen exposure (columns). As with **(A)**, there was strong support in the data for an interaction between day and habitat on beta diversity trajectories over time.

All individuals had similar bacterial species richness on Day 0 when they entered the experimental habitats, but the dynamics of host microbiome bacterial species difference over time differed markedly depending on habitat treatment (Figure 3A). The best supported model explaining differences in richness contained an interaction between day^2^ and habitat. When marginalizing the effects of time (sampling day) and variation among individuals in their change in diversity over time, individuals in complex habitats had greater skin bacterial diversity than those in simple habitats (Supplementary Figure S3). The top model explained 18.91% of variation in alpha diversity (95% credible interval 7.38–32.17%). There was no evidence that ranavirus exposure altered the dynamics of richness over time (Supplementary Tables S6, S7). When considering only the pre-exposure (Day 13) and post-exposure (Day 16) time points, the modal response was an increase in richness across the two time points. The top model examining factors predicting microbial community evenness contained only effects of day and day^2^, but no habitat main effect or interactions. The null model was also retained in the top model set (Supplementary Table S8). These data corroborate those from experiment 1 indicating a change in evenness over time. However, it is the environment (habitat) that appears to drive changes predominantly in the dynamics of microbial richness of amphibian skin over time.

#### Beta Diversity

PERMANOVA performed on Bray–Curtis distances revealed significant effects of habitat, day and a habitat:day interaction (all *p* = 0.001) on bacterial beta diversity (Figure 3B and Supplementary Table S9). Collectively these terms explained roughly 15% of the variation in variation among individuals in bacterial community structure. There was no evidence that exposure to ranavirus modified the structure of bacterial communities (all interaction terms containing an effect of ranavirus exposure, *p* > 0.05), nor evidence of a ranavirus main effect (*p* = 0.28, Supplementary Table S9). Linear modeling of factors predicting NMDS1 revealed clear evidence of habitat-dependent variation in beta diversity trajectory over time for all individuals (Figure 3C). The only model in the top model set explaining predictors of NMDS1 contained an interaction between habitat type and day^2^ (Figure 3C and Supplementary Figure S4), corroborating the results of the PERMANOVA above.

#### Functional Traits

Predicted functional analysis using PIPHILLIN revealed distinct separation in the functional repertoires of the amphibian skin bacterial microbiome based on habitat after 7 days (CCA analysis, effect of habitat *F*_(__1_,_45__)_ = 3.15, *p* = 0.01, Supplementary Figure S5). Analysis using DESeq2 revealed 12 pathways that were significantly more abundant in simple Habitats, and 8 pathways more abundant in complex habitats (Supplementary Table S10).

#### Viral Load Data

Viral loads of frogs following exposure to ranavirus in Experiment 2 were weak; mean viral load was 0.0013 viral copies per host cell [range 0.0001–0.01]. There was no significant difference in the mean viral load between animals in Complex and Simple habitats (Wilcoxon rank sum test, *W* = 88, *p* = 0.19, Supplementary Figure S6). No control animals in either habitat treatment had detectable levels of virus (Supplementary Figure S5).

### Patterns of Microbiome Stability Varied by Habitat (Experiment 2)

**FIGURE 4:**
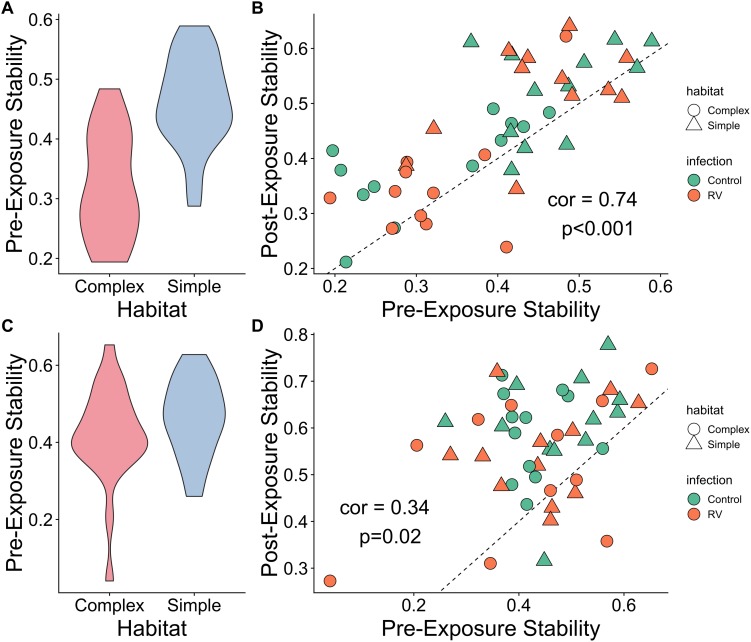
Microbiome stability over time. **(A)** Microbiome stability, measured as the correlation between ASV abundances across two times points, prior to pathogen exposure. Frogs in Simple habitats appear to have more stable microbial communities than those in Complex Habitats. **(B)** Scatterplot of microbiome stability over two sampling points prior to pathogen exposure (*x* axis) and two sampling points either side of pathogen exposure (*y* axis). Individual microbiome stability appears relatively consistent over time, irrespective of habitat or pathogen exposure. Dashed line represents 1:1 line of perfect correlation. Plots **(C,D)** are identical to plots **(A,B)**, but use only the top 100 most abundant ASVs for each habitat type, representing a “core microbiome.” There is no difference between habitat types in stability for the core microbiome **(C)**, and the correlation between stability values over time remains, though the relationship is weaker **(D)**.

When considering all ASVs, complex habitats exhibited decreased stability over time prior to infection when compared to simple habitats (*t* = 5.8, df = 43.3, *p* < 0.001; Figure 4A). However, an individual’s microbiome stability appeared consistent over time when comparing stability prior to pathogen exposure and stability following pathogen exposure (Figure 4B and Supplementary Figure S7A).

When considering only the top 100 ASVs, the difference in stability over time pre-infection was no longer apparent (*t* = 1.2, df = 42, *p* = 0.21; Figure 4C). Forty-seven ASVs from five Phyla were common to both sets of 100 most abundant ASVs by habitat (Supplementary Table S11). The most common Phylum of shared ASVs was *Proteobacteria*, comprising 32 of the 47 ASVs (68%). *Actinobacteria* and *Bacteroidetes* accounted for 13% each of the shared ASV taxonomy. At the genus level, notable shared ASVs were classified as *Citrobacter, Acinetobacter, Chryseobacterium*, and *Stenotrophomonas*, all of which have been associated with production of metabolites that inhibit other amphibian pathogens like *B. dendrobatidis* (e.g., Antwis and Harrison, 2018).

Both habitats still exhibit consistent levels of stability either side of exposure to the pathogen (Figure 4D and Supplementary Figure S7B), though the correlation is weaker. There was no evidence that habitat treatment, pathogen exposure or their interaction affected the magnitude of change in stability over time, for either all ASVs or the analysis restricted to the top 100 most abundant ASVs (ANOVA, all *p* > 0.27). Individual trajectories of microbiome richness and beta diversitty are provided in Supplementary Figures S8, S9.

## Discussion

Though our knowledge of the factors shaping the structure of the host-associated microbiota is increasing, studies directed at understanding the predictors of longitudinal variation of the microbiome in non-model organisms are relatively scarce (e.g., Smith et al., 2015; Videvall et al., 2019). Our results from two experiments suggest that the structure and temporal dynamics of the amphibian skin microbiome are influenced by both the environment and exposure to a lethal pathogen. Overall alpha diversity of the microbiome appeared to influence temporal stability, where more “species-rich” microbial communities were less stable over time compared to less diverse communities. Crucially, this effect disappeared when considering only the top 100 most abundant bacterial taxa, suggesting “core microbiome” stability may be relatively uniform irrespective of total diversity. Finally, our survival data suggest that higher skin microbiome diversity may correlate with greater survival following exposure to the lethal pathogen ranavirus. Our results have important implications for our understanding of factors driving variation among individuals in the stability of both their microbiomes and the strength of host-microbe interactions over time, and in turn how both traits may be compromised by external stressors such as exposure to pathogens.

### Environmental Microbial Diversity Influence Temporal Microbiome Dynamics

By manipulating the microbial reservoir in the environment, we elicited differential patterns of microbiome diversity on the skin of common frogs. Microbial communities differing in diversity also exhibited distinctive signatures of change over time. Higher diversity skin microbiomes appeared less stable over time, an effect driven primarily by weak correlations over time in the abundances of rarer bacterial taxa. The “core microbiome” of the most abundant ASVs in each habitat type appeared stable irrespective of overall diversity. Most strikingly, microbiome stability itself appeared conserved over time: stability between the first two time points correlated strongly with the two time points bracketing exposure to the pathogen. Our data therefore support the idea of *consistent* variation among individuals in microbiome stability over time, where structure itself is a function of the environment that an individual inhabits. These data support previous work indicating that environmental context and complexity is a key determinant of the assembly and stochasticity dynamics of host microbiomes, with important consequences for host resistance ot disease (Becker et al., 2017). It is notable that 47 of the 100 most abundant per-habitat ASVs were common to both habitats, alluding to a constrained core microbiome structure irrespective of habitat microbial diversity and structure.

A major outstanding question is what are the consequences of consistent within and among-individual variation in microbiome stability over time? Predicted functional analysis highlighted that differences in overall microbial community diversity also reflected differences in functional repertoire (see also Bletz et al., 2016) which could reflect variation in the presence or strength of key interactions between microbes and hosts such as production of antimicrobial metabolites that defend the host from pathogens (e.g., Brucker et al., 2008; Becker et al., 2009; Antwis and Harrison, 2018). Measures of temporal stability or stochasticity of microbiomes, and the processes that drive these traits, are critical for understanding the consistency of microbe-mediated functions over time. Most of the data we have on *within-individual* microbiome dynamics and stability come from human or model organism studies (e.g., Fink et al., 2013; Kelly et al., 2016; Gilbert et al., 2018; Schirmer et al., 2018), with relatively few on non-model organisms (e.g., Antwis et al., 2019; Videvall et al., 2019), and fewer still from controlled experiments (Kueneman et al., 2016; Grottoli et al., 2018). Though several studies have measured seasonal dynamics of microbiome in species such as aphids (Smith et al., 2015) and mosquitoes (Novakova et al., 2017); they rely on population-based metrics of microbiome structure that may mask substantial among-individual variation in microbiome dynamics. Amphibians and their skin microbiomes provide a model for understanding the processes shaping the forces of colonization, competition and coexistence of microbial species on a vertebrate host, and quantifying the emergent functional properties of these microbial communities and their consequences for the host. The properties of this system make it well suited to testing the applicability of established ecological theory derived from eukaryotic communities to prokaryotic assemblages associated with animals, plants and soils, including the relationship between biodiversity and ecosystem function (e.g., see Koskella et al., 2017; Greenspan et al., 2019).

### Exposure to Ranavirus Disrupts the Host Skin Microbiome

Our data from experiment 1 revealed that exposure to ranavirus elicited subtle but significant changes to the structure of the amphibian skin microbiome after 48 h. We predicted that more diverse microbial communities should be more resistant to perturbation by the ranavirus, but our data suggest that the skin microbiomes of individuals in both habitat treatments were affected by the pathogen. This supports previous work showing that pathogens like *Bd* can destabilize host microbiomes (e.g., Jani and Briggs, 2014; Walke et al., 2015; Longo and Zamudio, 2017). Though we didn’t detect a similar time:habitat:pathogen interaction in Experiment 2, this can be explained by the relatively low infection burdens in this experiment. Results from our experimental work here are well supported by counterpart investigations into the structure of the microbiota of wild common frogs. These studies have illustrated distinct differences in bacterial community structure at sites suffering mass mortality events due to ranavirus compared to sites where no such outbreaks have been detected (Campbell et al., 2018b, 2019), even after accounting for differences among populations (Campbell et al., 2019). These correlative data from wild frogs could represent bacterial communities in some populations associated with protection of the host from viral infection, or marked shifts in microbiome structure in populations suffering ranaviral infection. Use of pesticides has been associated with increased prevalence of ranaviruses (North et al., 2015) and could theoretically be mediated by disruption of the both environmental and host-associated bacterial communities. Considering these data with results from our experiments hints that both processes may be responsible for the patterns observed in nature. Disruption of the host microbiome by pathogens of wild vertebrates is likely to be far more common than the existing literature suggests. The scarcity of studies directed at quantifying microbiome disruption by pathogens means we currently lack the ability to compare the magnitude of the perturbation effect among host species and both host and pathogen taxonomic groups.

### Links Between Microbiome and Survival Following Ranavirus Exposure

Our controlled infection experiment revealed that individuals with less diverse microbiomes exhibited higher mortality following exposure to ranavirus compared to individuals with higher diversity microbial communities, consistent with our predictions. In our models, greater resistance to pathogenic infection as an emergent property of microbiome diversity would be evidenced by a diversity (habitat) by pathogen exposure interaction term. We note that though there was reasonable support for a model containing this interaction in our top model set, it was not retained under the nesting rule. As such, there exists some model section uncertainty regarding the effect of microbiome diversity on resistance to ranavirus infection. Several studies have provided evidence consistent with a correlation between overall microbiome diversity and susceptibility to infectious disease and costs associated with host responses to pathogen exposure (e.g., Cariveau et al., 2014; Kueneman et al., 2016), though these effects are not always consistent (e.g., Becker et al., 2019; Ma et al., 2019). Disruption of the normal microbiome by administration of antibiotics to laboratory mice can permit successful infection of *Clostridium difficile* (Theriot et al., 2014), loss of microbiome diversity in amphibians can increase susceptibility to the fungal pathogen *Bd* (Kueneman et al., 2016), and disruption of the microbiome in early life can increase downstream susceptibility to parasites (Knutie et al., 2017). Notably, augmentation of low diversity skin microbiomes with key taxa from the more diverse wild-type microbiome can reverse the observed increase in susceptibility to a lethal pathogen like *Bd* (Kueneman et al., 2016). Our habitat treatments differed in overall physical structure as well as microbial diversity, as complex habitats contained different terrestrial substrate as well as leaf litter in the water. Traits such as host microbiome richness, temporal dynamics, and resistance to disease will be governed by both extrinsic processes (microbial diversity present in the environment capable of colonizing the host) and intrinsic factors, such as host immunogenetic variation (e.g., Bolnick et al., 2014) and physiological stress (e.g., Lokmer and Wegner, 2015). Though variation in host stress due to structural heterogeneity between habitat treatments could have influenced microbiome dynamics, we believe this effect would be minimal in our data as all individuals were reared under captive conditions as tadpoles, and so were acclimated to conditions found in the simple habitat treatments. Nevertheless, future work will standardize the environmental structure of the habitats and manipulate only the microbial reservoir to remove the potential for such differences between treatments.

The mechanisms underpinning diversity-disease relationships in amphibians warrant further investigation. Microbiome diversity alone cannot be considered a beneficial trait for hosts; rather diversity itself is an emergent property of ecological processes playing out within the host (Shade, 2017) that underpin the true mechanism. More diverse microbiomes could be more likely to contain species producing antiviral compounds such as bacteriocins (see Drider et al., 2016), or to prime the host immune system to produce anti-microbial peptides (Woodhams et al., 2019) that can inactivate ranavirus virions (Chinchar et al., 2004). As expected, ranavirus-exposed individuals that died during the experiment had higher viral loads than those that survived. Higher survival in individuals with more diverse microbiomes could represent microbe-mediated defense preventing infection burdens from reaching lethal thresholds. Indeed, our predicted functional analysis of skin bacterial microbiomes revealed distinct differences dependent on diversity. Though complex skin microbiomes were predicted to differ in relative abundance of pathways linked to human viral infections, the relevance of such differences to amphibian defense against ranavirus remains to be determined. An important priority for future work is to quantify the true functional genetic repertoire of amphibian skin microbiomes to permit identification of potential metabolic pathways linked to disease, and examine how their relative abundance changes in concert with overall microbiome diversity and microbial species composition. Addressing this knowledge gap requires integration of further ‘omic tools such as shotgun metagenomics and metabolomics with more common amplicon sequencing metagenetics (Rebollar et al., 2016). Finally, Warne et al. (2019) recently showed that disruption of the gut microbiome in early life can influence host metabolism and susceptibility to ranavirus in later life. Given that the oral cavity and alimentary canal are major routes of infection for ranaviruses (e.g., Robert et al., 2011; Saucedo et al., 2019), one possibility is that measurements of skin microbiome diversity in amphibians are also reflective of gut microbiome diversity. Future work should quantify this covariation between multi-site microbiome dynamics and seek to understand the functional consequences of increased skin and gut microbiome diversity in hosts vulnerable to ranavirus.

## Author’s Note

This manuscript has been released as a preprint at bioRxiv (Harrison et al., 2017).

## Data Availability Statement

R markdown scripts and data sets permitting full reproduction of all analyses are hosted on FigShare at https://doi.org/10.6084/m9.figshare.c.4607198. Sequences have been uploaded to the NCBI Sequence Read Archive under BioProject accession numbers PRJNA559513 (Experiment 1) and PRJNA559522 (Experiment 2).

## Ethics Statement

The animal study was reviewed and approved by the Institute of Zoology Ethics Committee.

## Author Contributions

XH and TG designed the experiment. XH, SP, WL, CS, and TG conducted the experiment. XH and KH sequenced the microbial data. SP and WL produced ranaviral infection load data. XH analyzed the data. XH, SP, and TG wrote the manuscript with input from all authors.

## Conflict of Interest

The authors declare that the research was conducted in the absence of any commercial or financial relationships that could be construed as a potential conflict of interest.

## Footnotes

1 https://www.gardenwildlifehealth.org/

2 http://mc-stan.org/
