# Supplementary material for: Diversity-Stability Dynamics of the Amphibian Skin Microbiome and Susceptibility to a Lethal Viral Pathogen
Source: Front Microbiol. 2019 Dec 20;10:2883. doi: 10.3389/fmicb.2019.02883 (PMC6951417; doi:10.3389/fmicb.2019.02883)
Supplement: Supplementary file 1 [file Table_1.DOCX]

**Extraction Protocol**

The Qiagen plate extraction protocol has been modified to include an additional digestion using mutanolysin and some minor modifications to the standard plate extraction protocol. This protocol is easiest when processing two full plates of extractions at a time due to the requirement to balance the centrifuge.

The process will take approximately eight hours to complete and should be completed in a single session.

Before you begin, remove swabs from freezer and organise a plate map to record the well location of samples. Set incubator to 37⁰C.

**Mutanolysin Digestion and Swab Sampling**

1. Prepare mutanolysin buffer (per sample: 2.16µl mutanolysin @ 25Ku/ml + 177.84µl H_2_O) for 192 samples in a reagent reservoir: 456.192µl mutanolysin + 37.559ml autoclaved water
2. Aliquot 180µl of buffer to each tube of two racks of Qiagen Collection microtubes using the electronic multichannel pipette
3. Insert the first swab into position A1 of the collection microtubes, using the scissors to cover the top of the well cut the end off of the swab as close to the tip as possible
4. Clean scissors after each swab using 5% Bleach solution
5. Continue to fill the racks of tubes leaving G12 for the mock community and H12 as a negative then seal with strip lids, put a round bottom plate on top and secure using multiple rubber bands
6. Incubate at 37⁰C for 30 minutes

**Qiagen Extraction**

1. Spin the collection tubes down and set the incubator to 56⁰C
2. Remove lids from the tubes and add 25µl of Proteinase K to each well
3. Add 180µl of Buffer AL (without added ethanol) to each collection tube
4. Replace lids on the tubes and re-seal with plates and rubber bands
5. Shake sealed plates vigorously up and down for 15 s and centrifuge allowing the centrifuge to reach 3000rpm then stop
6. Incubate for one hour at 56⁰C, agitating mixture after 30 mins (make sure lids are still sealed)
7. Centrifuge the plates allowing the centrifuge to reach 3000rpm then stop
8. Carefully remove the caps and add 200µl of ethanol (96-100%)to each tube
9. Seal the tubes as above and shake vigorously up and down for 15s, centrifuge to 3000rpm
10. Place two DNeasy 96 plates on top of S-Blocks, label DNeasy plates
11. Remove the strip caps from the microtubes one at a time and carefully transfer the lysis mixture to the corresponding well of the DNeasy plates (carefully pipette from round the side of the swab and try to remove all the liquid from the tube, aspirating 650µl dispensing 600µl, set the aspirate speed of the pipette to 2 and the dispense to 3 to reduce frothing)
12. Continue to transfer both plates of lysis mixture and seal the DNeasy plates with AirPore Tape Sheets
13. Centrifuge for 10 mins at 3700rpm (for tube extractions 8000rpm for 1 minute)
14. Check all the liquid has passed through the filter, if not re-centrifuge
15. Remove the tape and add 500µl of Buffer AW1 (with ethanol added) to each sample
16. Seal the plates with AirPore Tape Sheet and centrifuge for 5 minutes at 3700rpm (for tube extractions 8000rpm for 1 minute)
17. Remove the tape and add 500µl of Buffer AW2 (with ethanol added) to each sample
18. Centrifuge for 30 mins at 3700rpm with no tape seal to make sure there is no residual ethanol (for tube extractions 14000 rom for 3 minutes)
19. Label 2 racks of Elution Microtubes
20. Place the DNeasy plate **in the correct orientation** on the rack of Elution Microtubes
21. Add 200µl of Buffer AE to each sample and seal with AirPore Tape Sheets, incubate at room temperature for 5 minutes
22. Centrifuge for 10 minutes at 4700rpm (for tube extractions 6000rpm for 1 minute)
23. Remove the DNeasy 96 plate from the Elution Microtubes and place in an empty P200 tip box, remove the sample form the Elution Microtubes and add it back on to the corresponding membrane of the DNeasy 96 plate, seal with an AirPore tape and incubate at room temperature for 5 minutes
24. Centrifuge for 10 minutes at 4700rpm
25. Before capping the Elution Microtubes take an aliquot plate to be used as a working plate of extractions.

**Indexed PCR Setup**

PCR’s are conducted using indexed primer sets to give a unique combination of barcodes to each sample. Three 10µl replicates are set up for each sample using HotFire POL master mix.

**Index Set Up**

Indexed primer sets

Set-A A7 and A5

Set-B A7 and B5

Set-C B7 and B5

Set-D B7 and A5

Set-A not to be run alone, index diversity is insufficient, for 96 or less samples use Set B, C, or D

Dilute relevant primer stocks to 10µM in strip tubes

Make a stock plate of indexed primers at 2µM

2µl of appropriate N5 index @ 10µM

2µl of appropriate N7 index @ 10µM

16µl PCR Water

**PCR Set Up**

1. Make a master mix of the taq and water

|  | Per Reaction | For 1 Plate | For 3 Plates |
| --- | --- | --- | --- |
| Hotfire-POL | 2µl | 224µl | 640µl (2*320µl) |
| PCR H_2_O | 4µl | 448µl | 1280µl (2*640µl) |

1. For 3 plates aliquot 230µl of MM into a strip of tubes, using a multichannel pipette add 6µl to each well of the PCR plate(s)
2. Aliquot 3µl of the 2µM primers into the corresponding wells of the PCR plates
3. Add 1µl of gDNA from the extraction aliquot plate to the PCR plates
4. Run the following PCR cycling conditions

| 95.0⁰C | - | 15 min |  |
| --- | --- | --- | --- |
| 95.0⁰C  50.0⁰C  72.0⁰C | -  -  - | 20s  1 min  1 min | x28 |
| 72.0⁰C | - | 10 min |  |
| 4.0⁰C | - | HOLD |  |

1. If you do not plan to run the gels immediately freeze the cycled PCR’s

**PCR check on agarose gel**

All three plates of PCR products are visualised on agarose gels

Gel Red Loading buffer: 350µl - 6x loading buffer, 250µl - H_2_O, 1.17µl - Gel Red

In the gel room:

1. Make 100ml of 2% agarose (100ml 0.5xTBE and 2g Agarose) and cool under the tap
2. Set up a gel tray with 4 28-tooth combs and pour the cooled gel mixture

In the Post-PCR genomics lab:

1. Add 26µl of Gel Red loading buffer to each well of a strip of 8 tubes
2. Using an 8-channel pipette add 2µl of Gel Red loading buffer to each well of a gel loading plate
3. Using an 8-channel pipette add 2µl of PCR product to the loading buffer and mix
4. In the strip of tubes containing Gel Red loading buffer make up 4 ladder samples, 2µl Gel Red loading buffer and 2µl ladder
5. Re-seal the PCR plate with a clear Qiagen seal and freeze

In the gel room:

1. Remove the dams from the gel tray and put the tray into one of the tanks before removing the combs
2. Load the full volume of sample and loading buffer using a multichannel pipette
3. Run the gel for 25 minutes at 120v 46mA 5w

**Pooling Replicates**

1. Use the gels to determine sample replicates that have failed
2. Remove plates from freezer and spin down
3. From replicate plate 1 remove and discard failed reactions
4. For replicate plate 2 mark the wells that have failed, remove the corresponding tips from a full tip box
5. Using a multichannel pipette transfer the full volume from the successful wells of replicate plate 2 into replicate plate 1
6. Repeat step 4 and 5 for replicate plate 3
7. At this point if the volume differs significantly from the volume you should have left from the PCR reactions make up the rest of the volume with PCR grade water

**Sample Clean-up**

Before you start:

The volume in the pooled plate should be 24µl

Bring the Ampure XP beads to room temperature (~30 minutes)

Make up 400µl of 70% ethanol for each sample to be cleaned

-for one plate, 35ml of Absolute Ethanol and 15ml of dd H_2_O

Prepare an empty tip box filled with tissue to discard liquid waste into

Add the beads to a reagent reservoir or strip of tubes depending on the number of samples to clean

1. Add 43µl of beads to each sample and pipette mix 10 times

- Incubate the plate at room temperature for 5 minutes

1. Put the plate on the magnetic stand for 2 minutes
2. Remove 62µl of supernatant and discard, avoid disturbing the beads

- If beads are also being aspirated it might be necessary to remove 55-60µl initially and remove the rest of the volume with a 10µl tip afterwards

1. With the plate still on the magnet add 200µl of 70% ethanol (from a reagent reservoir) without disturbing the beads

- Incubate for 30 seconds

1. Remove and discard the supernatant
2. Repeat steps 4-5 for a total of two washes
3. Incubate the plate for 5 minutes at room temperature to dry the beads

- Using a 10µl pipette remove and discard any remaining ethanol from each sample

1. Remove the plate from the magnet, add 40µl of PCR grade water to each well and pipette up and down 10 times to re-suspend the beads

- Incubate at room temperature for 2 minutes

1. Put the plate back onto the magnet and incubate for 1 minute or until the solution has cleared
2. Transfer 35µl of the clean product to a new plate and seal it with a Qiagen tape seal

**MiSeq Library Quantification Run**

Before you start:

Remove the TapeStation and Qubit ds High Sensitivity reagents from the fridge to come to room temperature for 30 minutes

**Sample Pooling**

1. Using a multichannel pipette aliquot 1µl of sample from the first column, paying close attention to the volume in each tip, and add to a strip of 8-tubes
2. Repeat this for the rest of the plate changing tips between columns
3. Pool the products from the strip of tubes into a 1.5ml tube
4. Repeat this process for the remaining plates to produce a single 1.5ml tube containing 1µl of each individual library

**Sample QC: Qubit**

1. Vortex and centrifuge Qubit reagents
2. In a 1.5ml tube prepare 5 reactions of master mix by adding 5µl of Qubit reagent to 995µl of sample buffer

- Vortex and briefly centrifuge

1. Add the following to 5 Qubit tubes

Tube-1 190µl of master mix, 10µl of standard 1

Tube-2 190µl of master mix, 10µl of standard 2

Tube-3 198µl of master mix, 2µl of pooled sample

Tube-4 198µl of master mix, 2µl of pooled sample

Tube-5 198µl of master mix, 2µl of pooled sample

1. Turn on the Qubit and select DNA - dsDNA HS assay
2. Select to read new standards
3. Follow on screen instructions to read standards and sample tubes
4. After reading the first sample tube select “calculate stock concentration”

- Change the sample volume to 2µl
- Change the units to ng/µl

1. Record the concentrations for the three sample replicates

**Sample QC: TapeStation**

1. Remove 2 tubes from a strip of TapeStation tubes and label 1 and 2
2. Add 3µl of sample buffer to each tube
3. To the first tube add 1µl of ladder
4. To the second tube add 1µl of pooled library sample
5. Seal the tubes and put onto the plate vortex for 60 seconds
6. Briefly spin down
7. Remove the lids, check liquid is still in the very bottom of the tube and put into the tube holder in the TapeStation
8. Insert a tape, and make sure the loading tip rack is full
9. In the TapeStation controller software select the first two wells of the tube holder and name the sample in the sample table
10. Start the run (save the run stating it is a library pool, your name and the date)
11. In TapeStation analysis click on “Electropeherogram” and select the sample lane, check the trace for signs of primer dimer

**MiSeq Loading**

If the concentration of the library pool is good and the TapeStation shows no sign of primer dimer the samples are ready to be denatured, diluted and loaded onto the MiSeq. This process will take approximately 90 minutes and must be completed in a single session. Before starting create a sample sheet using Illumina Experiment Manager (correctly arranged sample names and indexes can be copied from the “Illumina_EM_Format” tab on the sample tracking sheet) and transfer it to the SampleSheet folder on the MiSeq (rename the file with the reagent cartridge barcode). Use the Library Dilution calculator on the “QC_Run_Dil.” tab of the sample tracking sheet to calculate dilution volumes for the process below.

If you are ready to go ahead and load the MiSeq remove the reagent cartridge from the freezer and set it aside in a room temperature water bath for 1 hour, making sure to observe the max fill line. Keep the tube of HT1 at room temperature until defrosted and then store on ice. Remove the library pool, sequencing primers and the 10nM PhiX to defrost at room temperature. Set a heat block to 96⁰C.

**Denature and Dilute Sample and PhiX**

1. Prepare a fresh dilution of 0.2N NaOH

- 400µl H_2_O + 100µl 1N NaOH

1. To a 1.5ml tube add 10µl of sample pool (or 1:10 sample pool if necessary) and 10µl of 0.2N NaOH
2. To a different 1.5ml tube add 2µl of 10nM PhiX, 3µl of H_2_O and 5µl of 0.2N NaOH
3. Vortex the library and PhiX tubes for 5 seconds and spin for 1 minute at 400rcf
4. Incubate the tubes for 5 minutes at room temperature
5. To the denatured library tube add 980µl of HT1
6. To the denatured PhiX tube add 990µl of HT1, now 20pM (20pM dilution can be stored at -20 for three weeks)
7. Vortex library and PhiX and centrifuge for 1 minute
8. Dilute the sample to 3.5pM using the volumes from the Library Dilution Template sheet
9. Dilute the PhiX to3.5pM by adding 175µl of 20pM PhiX to 825µl of HT1
10. Invert library and PhiX tube to mix and pulse centrifuge
11. Put 950µl of 3.5pM library in a 1.5ml tube and add 50µl of 3.5pM PhiX, put this sample on ice

**Prepare Reagent Cartridge**

1. Remove the cartridge from the water bath and dry, tap on paper towel to remove as much water as possible from the base
2. Invert cartridge 10 times to mix reagents and inspect the reservoirs to make sure they are fully defrosted
3. Tap on the bench to make sure there are no bubbles at the bottom of the tubes
4. Using a 1000µl pipette tip pierce the foil of wells 12, 13 and 14
5. Place 3µl of 100mM Read-1 sequencing primer into a PCR tube
6. Using an extended length 200µl tip on a pipette set to 100µl take the primer from the PCR tube and add to well 12 of the cartridge, pipette mix 10 times
7. Repeat step 5 and 6 for the Index Primer and Read 2 sequencing primer

- Index Primer into well 13
- Read-2 Sequencing Primer into well 14

1. Set aside the MiSeq cartridge until the library is ready to Load

**Load Flow Cell**

1. On the MiSeq press sequence
2. Remove the flow cell and PR2 reagent box from fridge
3. Carefully remove flow cell from the buffer and rinse thoroughly with dd H_2_O, dry using Kimwipes paying particular attention to the edges of the glass
4. Wet another Kimwipe with absolute ethanol and clean the glass slide on both sides, avoid contact with the rubber gasket
5. Check the flow cell has no smudges or fibres from the wipes
6. Follow the instructions on the MiSeq to load the flow cell and PR2 buffer bottle, empty and replace the waste bottle

**Heat Denature Sample**

1. Using the heat block incubate the combined Library and PhiX tube for 2 minutes at 96⁰C
2. After the incubation invert the tube 2 times to mix and place in ice water bath for 5 minutes

**Load MiSeq Cartridge**

1. Using a 1000µl pipette tip pierce well 17 on the reagent cartridge
2. Load 600µl of the heat denatured Library / PhiX pool in to the cartridge
3. Load the cartridge into the MiSeq and wait for it to read the RFID
4. Follow on screen instructions, check the cycle numbers for the sequencing reads and the indexes
5. Wait for the MiSeq to complete its pre-run checks and then press start

**Post-Run Bleach Wash**

1. Select “Next” on the MiSeq and then “Perform Wash”
2. Remove the waste bottle and empty into the hazardous waste bottle by the MiSeq, return the empty bottle to the instrument
3. Remove the used buffer bottle and flush remnants down the sink with plenty of water
4. Remove the used reagent cartridge and put back into original box next to the MiSeq
5. Make up a fresh 10% Tween 20 solution by adding 5ml 100% Tween20 to 45ml dH_2_O, invert to mix
6. Add 25ml Tween 20 to 475ml dH_2_O to make a 0.5% Tween 20 Wash solution
7. Add 6ml of wash solution to each reservoir well except 17
8. Add 350ml of wash solution to the wash bottle
9. Prepare the bleach by adding 20µl of 6% Sodium Hypochlorite to 580µl of ddH_2_O to make a 0.2% solution
10. In the spare reagent cartridge tube add 50µl of 0.2% bleach to 950µl ddH_2_O to produce 1ml of 0.01% sodium Hypochlorite solution, load this tube into position 17 of the wash cartridge
11. Insert the cartridge and the wash bottle
12. Select perform template line wash and start the wash procedure
13. When the Post-Run Wash is complete press “Home” then “Perform Wash” and select “Post-Run Wash” but do not select preform template line wash
14. Remove the cartridge and top up the wells, remove the tube containing the bleach solution and add 6ml of wash buffer to position 17
15. Once completed the wash bottle, cartridge and flow cell must remain in place

**Collect Index Representation Information**

1. Open “Illumina Sequencing Analysis Viewer” and navigate to the latest run
2. Click on the Indexing tab and copy the table into excel
3. In the sample tracking sheet calculate the volume of each sample to add to a normalised pool

Volume of library x = (% reads identified/Number of samples loaded)

% Reads Identified for library x

- Depending on the variation in sample representation this might result in volumes that are not appropriate for pipetting, these samples might need a different pooling strategy

**MiSeq Sequencing Run**

From the quantification MiSeq run take the information from the index representation table to calculate the volume to add to the final sequencing pool. It might be necessary to make multiple pools for the variation in concentration and then pool these proportionally in the final pool.

**Sample Pooling**

1. Defrost all 4 clean pooled library plates and spin down
2. Pool the samples into a 1.5ml tube using the volumes calculated, start each plate with a full box of tips to track progress as well as the spreadsheet
3. Calculate the average sample volume being added to the pool and add that volume of the negative samples

**Sample QC: Qubit**

1. Vortex and centrifuge Qubit reagents
2. In a 1.5ml tube prepare 5 reactions of master mix by adding 5µl of Qubit reagent to 995µl of sample buffer

- Vortex and briefly centrifuge

1. Add the following to 5 Qubit tubes

Tube-1 190µl of master mix, 10µl of standard 1

Tube-2 190µl of master mix, 10µl of standard 2

Tube-3 198µl of master mix, 2µl of pooled sample

Tube-4 198µl of master mix, 2µl of pooled sample

Tube-5 198µl of master mix, 2µl of pooled sample

1. Turn on the Qubit and select DNA - dsDNA HS assay
2. Select to read new standards
3. Follow on screen instructions to read standards and sample tubes
4. After reading the first sample tube select “calculate stock concentration”

- Change the sample volume to 2µl
- Change the units to ng/µl

1. Record the concentrations for the three sample replicates

**MiSeq Loading**

This process will take approximately 90 minutes and must be completed in a single session. The sample sheet used for the library quantification run can be re-named with the 500 cycle cartridge barcode. Use the Library Dilution calculator on the “MiSeq_Run_Dil.” tab of the sample tracking sheet to calculate dilution volumes for the process below resulting in a 3.5pM library pool.

If you are ready to go ahead and load the MiSeq remove the reagent cartridge from the freezer and set it aside in a room temperature water bath for 1 hour, making sure to observe the max fill line. Keep the tube of HT1 at room temperature until defrosted and then store on ice. Remove the library pool, sequencing primers and the 10nM PhiX to defrost at room temperature. Set a heat block to 96⁰C.

**MiSeq Loading**

**Denature and Dilute Sample and PhiX**

1. Prepare a fresh dilution of 0.2N NaOH

- 400µl H_2_O + 100µl 1N NaOH

1. To a 1.5ml tube add 10µl of sample pool (or 1:10 sample pool if necessary) and 10µl of 0.2N NaOH
2. To a different 1.5ml tube add 2µl of 10nM PhiX, 3µl of H_2_O and 5µl of 0.2N NaOH
3. Vortex the library and PhiX tubes for 5 seconds and spin for 1 minute at 400rcf
4. Incubate the tubes for 5 minutes at room temperature
5. To the denatured library tube add 980µl of HT1
6. To the denatured PhiX tube add 990µl of HT1, now 20pM (20pM dilution can be stored at -20 for three weeks)
7. Vortex library and PhiX and centrifuge for 1 minute
8. Dilute the sample to 3.5pM using the volumes from the Library Dilution Template sheet
9. Dilute the PhiX to3.5pM by adding 175µl of 20pM PhiX to 825µl of HT1
10. Invert library and PhiX tube to mix and pulse centrifuge
11. Put 950µl of 3.5pM library in a 1.5ml tube and add 50µl of 3.5pM PhiX, put this sample on ice

**Prepare Reagent Cartridge**

1. Remove the cartridge from the water bath and dry, tap on paper towel to remove as much water as possible from the base
2. Invert cartridge 10 times to mix reagents and inspect the reservoirs to make sure they are fully defrosted
3. Tap on the bench to make sure there are no bubbles at the bottom of the tubes
4. Using a 1000µl pipette tip pierce the foil of wells 12, 13 and 14
5. Place 3µl of 100mM Read-1 sequencing primer into a PCR tube
6. Using an extended length 200µl tip on a pipette set to 100µl take the primer from the PCR tube and add to well 12 of the cartridge, pipette mix 10 times
7. Repeat step 5 and 6 for the Index Primer and Read 2 sequencing primer

- Index Primer into well 13
- Read-2 Sequencing Primer into well 14

1. Set aside the MiSeq cartridge until the library is ready to Load

**Load Flow Cell**

1. On the MiSeq press sequence
2. Remove the flow cell and PR2 reagent box from fridge
3. Carefully remove flow cell from the buffer and rinse thoroughly with dd H_2_O, dry using Kimwipes paying particular attention to the edges of the glass
4. Wet another Kimwipe with absolute ethanol and clean the glass slide on both sides, avoid contact with the rubber gasket
5. Check the flow cell has no smudges or fibres from the wipes
6. Follow the instructions on the MiSeq to load the flow cell and PR2 buffer bottle, empty and replace the waste bottle

**Heat Denature Sample**

1. Using the heat block incubate the combined Library and PhiX tube for 2 minutes at 96⁰C
2. After the incubation invert the tube 2 times to mix and place in ice water bath for 5 minutes

**Load MiSeq Cartridge**

1. Using a 1000µl pipette tip pierce well 17 on the reagent cartridge
2. Load 600µl of the heat denatured Library / PhiX pool in to the cartridge
3. Load the cartridge into the MiSeq and wait for it to read the RFID
4. Follow on screen instructions, check the cycle numbers for the sequencing reads and the indexes
5. Wait for the MiSeq to complete its pre-run checks and then press start

**Post-Run Bleach Wash**

1. Select “Next” on the MiSeq and then “Perform Wash”
2. Remove the waste bottle and empty into the hazardous waste bottle by the MiSeq, return the empty bottle to the instrument
3. Remove the used buffer bottle and flush remnants down the sink with plenty of water
4. Remove the used reagent cartridge and put back into original box next to the MiSeq
5. Make up a fresh 10% Tween 20 solution by adding 5ml 100% Tween20 to 45ml dH_2_O, invert to mix
6. Add 25ml Tween 20 to 475ml dH_2_O to make a 0.5% Tween 20 Wash solution
7. Add 6ml of wash solution to each reservoir well except 17
8. Add 350ml of wash solution to the wash bottle
9. Prepare the bleach by adding 20µl of 6% Sodium Hypochlorite to 580µl of ddH_2_O to make a 0.2% solution
10. In the spare reagent cartridge tube add 50µl of 0.2% bleach to 950µl ddH_2_O to produce 1ml of 0.01% sodium Hypochlorite solution, load this tube into position 17 of the wash cartridge
11. Insert the cartridge and the wash bottle
12. Select perform template line wash and start the wash procedure
13. When the Post-Run Wash is complete press “Home” then “Perform Wash” and select “Post-Run Wash” but do not select preform template line wash
14. Remove the cartridge and top up the wells, remove the tube containing the bleach solution and add 6ml of wash buffer to position 17
15. Once completed the wash bottle, cartridge and flow cell must remain in place

**Save Fastq Files**

1. On the MiSeq locate your run folder

D:/Illumina/MiSeq_Output/ <Run_Folder>/Data/Intensities/Basecalls

1. Copy R1 and R2 fastq files for each sample to an external hard drive (this will be a slow process due to screening of files by anti-virus software)

Yuan S, Cohen DB, Ravel J, Abdo Z, Forney LJ (2012) Evaluation of Methods for the Extraction and Purification of DNA from the Human Microbiome. PLoS ONE 7(3): e33865. doi:10.1371/journal.pone.0033865
