# Supplementary material for: Diversity-Stability Dynamics of the Amphibian Skin Microbiome and Susceptibility to a Lethal Viral Pathogen
Source: Front Microbiol. 2019 Dec 20;10:2883. doi: 10.3389/fmicb.2019.02883 (PMC6951417; doi:10.3389/fmicb.2019.02883)
Supplement: Supplementary file 2 [file Table_2.docx]

Supplementary Information

## Diversity-Stability Dynamics of the Amphibian Skin Microbiome and Susceptibility to a Lethal Viral Pathogen

## XA Harrison et al.

### FIGURE S1.

Relationship between alpha diversity of environment and frog skin, measured as effective number of species (exponent of Shannon diversity). There was a strong correlation between the bacterial diversity of the habitat (experimental block) and mean bacterial diversity of individuals within a block (r=0.82, p=0.001). Trend line from a linear fit for illustration.

### FIGURE S2

Data from Experiment 1 illustrating the differences in microbial community evenness due to time (2 = pre-exposure; 3 = post exposure to ranavirus) and habitat (simple or complex). Circles represent individuals in complex habitats, triangles those in simple habitats. Points are coloured by ranavirus exposure. The dominant effect in the data was that evenness appeared to be higher in complex habitat treatments, but this effect operated via an interaction with time as was most apparent at time point 3. Though there was some support for an interaction with ranavirus exposure, neither this term nor the time:habitat yerm were in all models in the top model set (See Table S2a,b).

### FIGURE S3

Predicted alpha diversity (effective number of species) depending on habitat treatment from Experiment 2. Estimates are from a GLMM, marginalising the effect of time and individual repeated measures, taken from the top model in Table S5. These data replicate the results of Experiment 1 demonstrating an increase in alpha diversity for individuals in Complex habitats.

### FIGURE S4.

Fitted means from the top model explaining variation in beta diversity (NMDS1) of Common Frog skin bacterial communities from Experiment 2. The model contained a a habitat*day^2^ interaction and all component nested terms, as well as random intercept term for individual frog ID (n=47 frogs). The model could not support a random slope for day and suffered convergence issues.


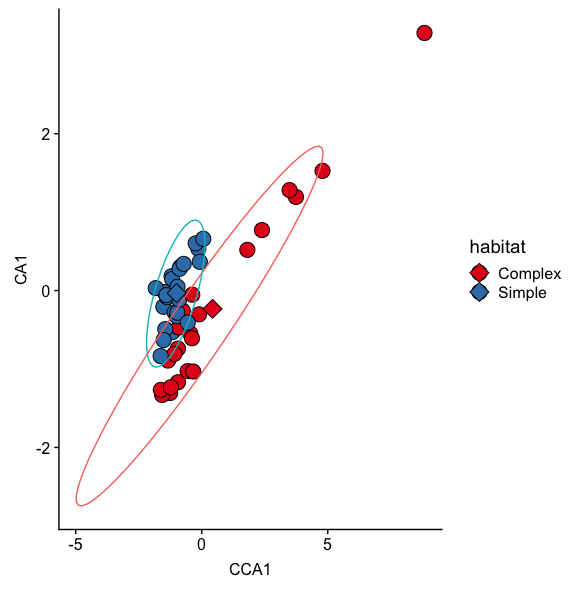


### FIGURE S5

Constrained Correspondence Analysis (CCA) plot of predicted functional pathways of amphibian skin microbiomes dependent on habitat after 7 days in habitat treatments. There was significant differentiation of functional profiles based on habitat (CCA analysis F_1,45_ = 3.15, p=0.01). Individual samples are represented by circles; group centroids by diamonds.

### FIGURE S6. VIRAL LOADS OF RANAVIRUS EXPOSED ANIMALS

Viral load data from Experiment 2 for ranavirus-exposed and control individuals in both Simple and Complex habitats, normalised to host DNA following Leung et al 2017. Viral loads were low compared to Experiment 1 (see Fig. 2B main manuscript). No Control individuals showed evidence of infection.

### FIGURE S7

Correlation between pre- and post-infection stability, split by habitat and disease exposure, for A) all ASVs and B) the top 100 most abundant ASVs in each habitat.

### FIGURE S8. TEMPORAL TRENDS IN ALPHA DIVERSITY BY INDIVIDUAL

Temporal trajectory of bacterial alpha diversity of 47 individuals over 4 sampling points. X-axis is time (sampling day) and Y-axis is effective number of species (exponent of Shannon diversity). Frogs differed markedly in their total bacterial alpha diversity depending on Habitat when marginalising with respect to time and among-individual variation. Habitat also modified the slope of the change in diversity over time (Habitat: Day Interaction; Table S5).

### FIGURE S9. TEMPORAL TRENDS IN BETA DIVERSITY BY INDIVIDUAL

Temporal trajectory of bacterial beta diversity (NMDS1) of 47 individuals over 4 sampling points. X-axis is time (sampling day) and Y-axis is primary axis of an NMDS ordination of bacterial communities (k=3, stress =12.6%). Frogs differed markedly in their total bacterial alpha diversity depending on Habitat when marginalising with respect to time and among-individual variation. Habitat also modified the slope of the change in diversity over time (Habitat: Day Interaction; see also Fig. S7).

| Parameter | Mean | Lower_95 | Upper_95 |
| --- | --- | --- | --- |
| Intercept (Complex Habitats, Pre-Infection) | 4.11 | 3.92 | 4.29 |
| Simple Habitats | -0.80 | -1.07 | -0.53 |
| Post Infection | 0.27 | 0.10 | 0.45 |
| Simple Habitats:Post Infection | -0.10 | -0.37 | 0.16 |

### TABLE S1

Model averaged parameter estimates from the top model set of models explaining variation in alpha diversity (richness measured as effective number of species) of Common Frog skin bacterial communities in Experiment 1. Microbial communities changed significantly in richness over time, and frogs in Simple habitats possessed lower alpha diversity than those in Complex habitats. Estimates are full model averages using the ‘zeroes’ method, accounting for when terms are absent from some models in the top model set.

| Intercept | Habitat | Ranavirus | Time | Habitat:Ranavirus | Habitat:Time | df | logLik | AICc | delta | weight |
| --- | --- | --- | --- | --- | --- | --- | --- | --- | --- | --- |
| 0.8426 | + | + | + | + | + | 8 | -43.223 | 103.3 | 0 | 0.537 |
| 0.9688 | + |  | + |  | + | 6 | -45.838 | 104.2 | 0.86 | 0.349 |
| 0.8961 | + | + | + | + |  | 7 | -46.335 | 107.3 | 4.03 | 0.072 |
| 1.024 | + |  | + |  |  | 5 | -49.02 | 108.4 | 5.08 | 0.042 |

TABLE S2a

AICc model selection output investigating factors predicting microbiome evenness in Experiment 1. Models shown are within delta-6 AICc units of the top model. We also applied the nesting rule to omit models that were more complex versions of models with better AIC support (lower AICc scores)

| **Parameter** | **Mean** | **Lower_95** | **Upper_95** |
| --- | --- | --- | --- |
| Intercept (Complex Habitats, Pre-Infection) | 0.90 | 0.71 | 1.09 |
| Habitat_Simple | -0.16 | -0.41 | 0.10 |
| Ranavirus Exposure | 0.15 | -0.13 | 0.44 |
| Time_3 | 0.27 | 0.13 | 0.41 |
| Habitat_Simple:Ranavirus_Exposure | -0.17 | -0.52 | 0.18 |
| Habitat_Simple:Time_3 | -0.20 | -0.42 | 0.02 |

### TABLE S2b

Model averaged estimates for factors predicting skin microbiome evenness on Common Frog skin.

| Effect | Df | SumsOfSqs | MeanSqs | F.Model | R2 | Pr(>F) |
| --- | --- | --- | --- | --- | --- | --- |
| box | 1 | 1.34 | 1.34 | 6.55 | 0.03 | 0.001 |
| habitat | 1 | 7.46 | 7.46 | 36.43 | 0.16 | 0.001 |
| infection | 1 | 1.08 | 1.08 | 5.28 | 0.02 | 0.001 |
| time | 1 | 1.31 | 1.31 | 6.42 | 0.03 | 0.001 |
| habitat:infection | 1 | 0.90 | 0.90 | 4.40 | 0.02 | 0.001 |
| habitat:time | 1 | 0.42 | 0.42 | 2.03 | 0.01 | 0.013 |
| infection:time | 1 | 0.34 | 0.34 | 1.65 | 0.01 | 0.044 |
| habitat:infection:time | 1 | 0.32 | 0.32 | 1.58 | 0.01 | 0.078 |
| Residuals | 164 | 33.57 | 0.20 | NA | 0.72 | NA |
| Total | 172 | 46.73 | NA | NA | 1.00 | NA |

### TABLE S3.

PERMANOVA output of factors explaining variation in beta diversity in amphibian skin microbial communities in Experiment 1.

| Habitat | Treatment | Habitat:  Treatment | df | logLik | AICc | delta | retained |
| --- | --- | --- | --- | --- | --- | --- | --- |
| + | + | - | 2 | -110.441 | 225.041 | 0.000 | Y |
| - | + | - | 1 | -111.596 | 225.258 | 0.218 | Y |
| + | + | + | 3 | -110.334 | 226.971 | 1.930 | N |

### TABLE S4

Mixed effect survival model output of factors explaining variation in survival based on habitat and exposure to ranavirus in Experiment 1. Only the top two models were retained under the nesting rule, as the model containing an interaction between habitat complexity and pathogen exposure was a more complex version of a model with better AIC support. ‘+’ indicates a term was present and ‘-‘ indicates that term was not included in a model.

| Intercept | Habitat | Mortality | Hab:Mort | df | logLik | AICc | delta | weight | retained |
| --- | --- | --- | --- | --- | --- | --- | --- | --- | --- |
| 2.996 |  | + |  | 4 | -223.72 | 456.40 | 0.00 | 0.53 | Y |
| 4.058 | + | + | + | 6 | -221.67 | 457.50 | 1.11 | 0.31 | N |
| 3.359 | + | + |  | 5 | -223.66 | 458.90 | 2.43 | 0.16 | N |
| 1.39 |  |  |  | 3 | -231.11 | 468.80 | 12.37 | 0.00 | N |
| 1.429 | + |  |  | 4 | -231.11 | 471.20 | 14.79 | 0.00 | N |

### TABLE S5

AICc model selection output from a mixed effects model explaining difference in viral load in ranavirus exposed-individuals in Experiment 1.

Habitat: Simple or Complex Habitats; Mortality: Died during the experiment following exposure vs survived. Only the top model was retained under the nesting rule. The null model and a model containing only habitat had a delta-AIC greater than 6.

| Intercept | Day | Day^2^ | Habitat | Pathogen | Hab:Day^2^ | df | logLik | AICc | delta | weight | Retained |
| --- | --- | --- | --- | --- | --- | --- | --- | --- | --- | --- | --- |
| 3.59 | 0.01 | 0.09 | + |  | + | 9 | -829.65 | 1678.30 | 0.00 | 0.36 | Y |
| 3.67 | 0.01 | 0.09 | + | + | + | 10 | -828.62 | 1678.50 | 0.16 | 0.33 | N |
| 3.44 | 0.00 | 0.23 | + |  |  | 8 | -831.55 | 1679.90 | 1.59 | 0.16 | Y |
| 3.51 | 0.00 | 0.23 | + | + |  | 9 | -830.62 | 1680.30 | 1.94 | 0.14 | N |
| 3.77 |  |  | + | + |  | 7 | -836.31 | 1687.20 | 8.92 | 0.00 | N |
| 3.70 |  |  | + |  |  | 6 | -837.40 | 1687.30 | 8.95 | 0.00 | N |
| 3.70 | -0.05 |  | + |  |  | 7 | -837.02 | 1688.70 | 10.34 | 0.00 | N |
| 3.77 | -0.05 |  | + | + |  | 8 | -835.95 | 1688.70 | 10.38 | 0.00 | N |
| 3.41 | 0.03 | 0.21 |  | + |  | 8 | -837.51 | 1691.80 | 13.51 | 0.00 | N |
| 3.34 | 0.03 | 0.20 |  |  |  | 7 | -838.73 | 1692.10 | 13.77 | 0.00 | N |
| 3.61 |  |  |  | + |  | 6 | -841.68 | 1695.80 | 17.51 | 0.00 | N |
| 3.54 |  |  |  |  |  | 5 | -842.88 | 1696.10 | 17.76 | 0.00 | N |
| 3.61 | -0.03 |  |  | + |  | 7 | -841.52 | 1697.70 | 19.34 | 0.00 | N |
| 3.54 | -0.03 |  |  |  |  | 6 | -842.69 | 1697.80 | 19.53 | 0.00 | N |

### TABLE S6

AICc model selection table from models set of models explaining variation in species richness of Common Frog skin bacterial communities from Experiment 2. All models contained a random slope for time (Day, n=4 time points per frog) given a random intercept for individual frog (n=47 frogs).

| Parameter | Mean | Lower_95 | Upper_95 |
| --- | --- | --- | --- |
| Intercept (Complex Habitats) | 3.54 | 3.25 | 3.83 |
| Day | 0.01 | -0.11 | 0.12 |
| Day^2 | 0.14 | -0.08 | 0.35 |
| Habitat_Simple | -0.51 | -0.92 | -0.10 |
| Habitat_Simple:Day^2 | 0.18 | -0.14 | 0.49 |

### TABLE S7

Model averaged parameter estimates from the 2 models retained from Table S6 (species richness). Estimates are full model averages using the ‘zeroes’ method, accounting for when terms are absent from some models in the top model set. All models contained a random slope for time (Day, n=4 time points per frog) given a random intercept for individual frog (n=47 frogs).

| Intercept | Day | Day^2^ | df | logLik | AICc | delta | weight |
| --- | --- | --- | --- | --- | --- | --- | --- |
| 0.3906 | -0.01238 | 0.1815 | 5 | -156.924 | 324.2 | 0 | 0.659 |
| 0.5712 |  |  | 3 | -159.685 | 325.5 | 1.32 | 0.341 |

### TABLE S8

Models in the delta-6 AICc model set investigating factors predicting microbiome *evenness* in Experiment 2. The top model contained an effect of day and quadratic effect of day. The null model was also retained. We applied the nesting rule to omit models that were more complex versions of models with better AIC support (lower AICc scores)

| Term | Df | SumsOfSqs | MeanSqs | F.Model | R2 | Pr(>F) |
| --- | --- | --- | --- | --- | --- | --- |
| habitat | 1 | 1.917 | 1.91718 | 10.6002 | 0.04975 | 0.001 |
| day | 1 | 2.563 | 2.56308 | 14.1714 | 0.06651 | 0.001 |
| infection | 1 | 0.202 | 0.20206 | 1.1172 | 0.00524 | 0.284 |
| habitat:day | 1 | 0.838 | 0.83813 | 4.6341 | 0.02175 | 0.001 |
| habitat:infection | 1 | 0.208 | 0.20825 | 1.1514 | 0.0054 | 0.293 |
| day:infection | 1 | 0.149 | 0.14941 | 0.8261 | 0.00388 | 0.596 |
| habitat:day:infection | 1 | 0.103 | 0.10264 | 0.5675 | 0.00266 | 0.916 |
| Residuals | 180 | 32.555 | 0.18086 | 0.8448 |  |  |
| Total | 187 | 38.536 | 1 |  |  |  |

### TABLE S9

PERMANOVA output of factors explaining variation of beta diversity of amphibian skin in Experiment 2. Significant terms are shown in bold.

| Pathway Code | log2FoldChange | Pathway |
| --- | --- | --- |
| ko01052 | -6.576903317 | Type I polyketide structures |
| ko00941 | -5.524986524 | Flavonoid biosynthesis |
| ko00945 | -5.524986524 | Stilbenoid, diarylheptanoid and gingerol biosynthesis |
| ko00532 | -2.882641168 | Glycosaminoglycan biosynthesis - chondroitin sulfate / dermatan sulfate |
| ko00534 | -2.882641168 | Glycosaminoglycan biosynthesis - heparan sulfate / heparin |
| ko00253 | -2.624142654 | Tetracycline biosynthesis |
| ko05169 | -2.085104007 | Epstein-Barr virus infection |
| ko00944 | -1.592103093 | Flavone and flavonol biosynthesis |
| ko01057 | -1.5571307 | Biosynthesis of type II polyketide products |
| ko04144 | -1.518640455 | Endocytosis |
| ko04973 | -0.846725677 | Carbohydrate digestion and absorption |
| ko00405 | -0.22329223 | Phenazine biosynthesis |
| ko04113 | 0.279712481 | Meiosis - yeast |
| ko04080 | 0.774831534 | Neuroactive ligand-receptor interaction |
| ko05166 | 0.781686694 | Human T-cell leukemia virus 1 infection |
| ko00100 | 1.617464475 | Steroid biosynthesis |
| ko05322 | 1.684552659 | Systemic lupus erythematosus |
| ko00363 | 1.870129134 | Bisphenol degradation |
| ko00902 | 5.103963219 | Monoterpenoid biosynthesis |
| ko00563 | 7.356621568 | Glycosylphosphatidylinositol (GPI)-anchor biosynthesis |

### TABLE S10.

Differentially abundant pathways from predicted functional analysis of Common frog skin bacterial communities after 7 days in habitat treatments (see Fig. S4). Negative log2foldChange values indicate pathways more abundant in Simple Habitat; positive values are more abundant in Complex habitats.

| ASV ID | Kingdom | Phylum | Class | Order | Family | Genus |
| --- | --- | --- | --- | --- | --- | --- |
| SV2 | Bacteria | Actinobacteria | Actinobacteria | Micrococcales | Sanguibacteraceae | Sanguibacter |
| SV3 | Bacteria | Proteobacteria | Gammaproteobacteria | Pseudomonadales | Pseudomonadaceae | Pseudomonas |
| SV5 | Bacteria | Proteobacteria | Gammaproteobacteria | Xanthomonadales | Xanthomonadaceae | Stenotrophomonas |
| SV7 | Bacteria | Proteobacteria | Betaproteobacteria | Burkholderiales | Burkholderiaceae | Limnobacter |
| SV9 | Bacteria | Proteobacteria | Gammaproteobacteria | Pseudomonadales | Moraxellaceae | Alkanindiges |
| SV14 | Bacteria | Proteobacteria | Gammaproteobacteria | Pseudomonadales | Pseudomonadaceae | Pseudomonas |
| SV16 | Bacteria | Bacteroidetes | Flavobacteriia | Flavobacteriales | Flavobacteriaceae | Chryseobacterium |
| SV18 | Bacteria | Proteobacteria | Betaproteobacteria | Burkholderiales | Alcaligenaceae | Verticia |
| SV19 | Bacteria | Proteobacteria | Gammaproteobacteria | Xanthomonadales | Nevskiaceae | Nevskia |
| SV20 | Bacteria | Proteobacteria | Betaproteobacteria | Burkholderiales | Comamonadaceae | Variovorax |
| SV22 | Bacteria | Proteobacteria | Gammaproteobacteria | Pseudomonadales | Moraxellaceae | Acinetobacter |
| SV24 | Bacteria | Proteobacteria | Gammaproteobacteria | Pseudomonadales | Moraxellaceae | Acinetobacter |
| SV26 | Bacteria | Proteobacteria | Gammaproteobacteria | Enterobacteriales | Enterobacteriaceae | Citrobacter |
| SV27 | Bacteria | Proteobacteria | Betaproteobacteria | Burkholderiales | Comamonadaceae | Variovorax |
| SV28 | Bacteria | Proteobacteria | Gammaproteobacteria | Enterobacteriales | Enterobacteriaceae | Klebsiella |
| SV31 | Bacteria | Proteobacteria | Gammaproteobacteria | Pseudomonadales | Pseudomonadaceae | Pseudomonas |
| SV32 | Bacteria | Bacteroidetes | Flavobacteriia | Flavobacteriales | Cryomorphaceae | NA |
| SV34 | Bacteria | Proteobacteria | Gammaproteobacteria | Pseudomonadales | Pseudomonadaceae | Pseudomonas |
| SV35 | Bacteria | Actinobacteria | Actinobacteria | Micrococcales | Microbacteriaceae | NA |
| SV37 | Bacteria | Proteobacteria | Betaproteobacteria | Burkholderiales | Comamonadaceae | NA |
| SV39 | Bacteria | Proteobacteria | Gammaproteobacteria | Pseudomonadales | Pseudomonadaceae | Pseudomonas |
| SV40 | Bacteria | Proteobacteria | Betaproteobacteria | Burkholderiales | Comamonadaceae | Limnohabitans |
| SV41 | Bacteria | Actinobacteria | Actinobacteria | Propionibacteriales | Nocardioidaceae | Nocardioides |
| SV46 | Bacteria | Firmicutes | Bacilli | Bacillales | Staphylococcaceae | Staphylococcus |
| SV48 | Bacteria | Proteobacteria | Gammaproteobacteria | Chromatiales | Chromatiaceae | Rheinheimera |
| SV52 | Bacteria | Proteobacteria | Gammaproteobacteria | Pseudomonadales | Pseudomonadaceae | Pseudomonas |
| SV53 | Bacteria | Firmicutes | Bacilli | Bacillales | Staphylococcaceae | Staphylococcus |
| SV57 | Bacteria | Actinobacteria | Actinobacteria | Micrococcales | Micrococcaceae | Paeniglutamicibacter |
| SV59 | Bacteria | Bacteroidetes | Bacteroidia | Bacteroidales | Bacteroidaceae | Bacteroides |
| SV61 | Bacteria | Proteobacteria | Gammaproteobacteria | Xanthomonadales | Solimonadaceae | Fontimonas |
| SV62 | Bacteria | Bacteroidetes | Flavobacteriia | Flavobacteriales | Flavobacteriaceae | Flavobacterium |
| SV64 | Bacteria | Proteobacteria | Betaproteobacteria | Burkholderiales | Comamonadaceae | Delftia |
| SV65 | Bacteria | Actinobacteria | Actinobacteria | Micrococcales | Brevibacteriaceae | Brevibacterium |
| SV67 | Bacteria | Proteobacteria | Betaproteobacteria | Methylophilales | Methylophilaceae | Methylophilus |
| SV70 | Bacteria | Proteobacteria | Gammaproteobacteria | Enterobacteriales | Enterobacteriaceae | Pantoea |
| SV71 | Bacteria | Proteobacteria | Betaproteobacteria | Burkholderiales | Comamonadaceae | Simplicispira |
| SV73 | Bacteria | Proteobacteria | Betaproteobacteria | Burkholderiales | Comamonadaceae | NA |
| SV78 | Bacteria | Proteobacteria | Alphaproteobacteria | Rhodobacterales | Rhodobacteraceae | Rhodobacter |
| SV84 | Bacteria | Proteobacteria | Betaproteobacteria | Neisseriales | Neisseriaceae | Vogesella |
| SV87 | Bacteria | Actinobacteria | Actinobacteria | Micrococcales | Microbacteriaceae | Curtobacterium |
| SV88 | Bacteria | Bacteroidetes | Flavobacteriia | Flavobacteriales | Cryomorphaceae | Fluviicola |
| SV89 | Bacteria | Proteobacteria | Gammaproteobacteria | Pseudomonadales | Pseudomonadaceae | Pseudomonas |
| SV92 | Bacteria | Proteobacteria | Betaproteobacteria | Burkholderiales | Comamonadaceae | Aquabacterium |
| SV93 | Bacteria | Proteobacteria | Gammaproteobacteria | Xanthomonadales | Xanthomonadaceae | Stenotrophomonas |
| SV104 | Bacteria | Proteobacteria | Betaproteobacteria | Burkholderiales | Oxalobacteraceae | Undibacterium |
| SV108 | Bacteria | Bacteroidetes | Sphingobacteriia | Sphingobacteriales | Sphingobacteriaceae | Sphingobacterium |
| SV148 | Bacteria | Verrucomicrobia | Verrucomicrobiae | Verrucomicrobiales | Verrucomicrobiaceae | Haloferula |

### TABLE S11.

Taxonomy of 47 ASVs common to the 100 most abundant ASVs in both complex and simple habitat
